# Comparison of CG, CKD-EPI[AS] and CKD-EPI[ASR] equations to estimate glomerular filtration rate and predict mortality in treatment naïve people living with HIV in Zimbabwe

**DOI:** 10.1186/s12882-023-03159-5

**Published:** 2023-05-08

**Authors:** Mitchell Hunter-Dickson, Douglas Drak, Matthew Anderson, Tinei Shamu, Cleophas Chimbetete, Rumbidzai Dahwa, David M. Gracey

**Affiliations:** 1 Department of Renal Medicine, Royal Prince Alfred Hospital, Camperdown, NSW Australia; 2 Central Clinical School, Faculty of Medicine, The University of Sydney, Sydney, Australia; 3 Newlands Clinic, Newlands, Harare, Zimbabwe; 4 Faculty of Medicine and Health Sciences, University of Zimbabwe, Harare, Zimbabwe

**Keywords:** HIV, Renal insufficiency, Zimbabwe, Screening

## Abstract

**Background:**

Renal impairment in people living with HIV (PWH) in Sub-Saharan Africa is common and associated with increased morbidity and mortality. The ideal equation to estimate glomerular filtration rate (eGFR) in this population remains unclear. That which best predicts clinical risk may be the most appropriate while validation studies are awaited. Here we compare the Cockcroft-Gault (CG), Chronic Kidney Disease Epidemiology Collaboration (CKD-EPI[ASR]) and the CKD-EPI equation with the race coefficient removed (CKD-EPI[AS]), in a population of anti-retroviral therapy (ART) naïve PWH in Zimbabwe to assess which equation best predicts mortality.

**Methods:**

A retrospective cohort study of treatment naïve PWH at the Newlands Clinic in Harare, Zimbabwe was completed. The study included all patients commencing ART between 2007 and 2019. Predictors of mortality were assessed by multivariable logistic regression.

**Results:**

A total of 2991 patients were followed-up for a median of 4.6 years. The cohort was 62.1% female, with 26.1% of patients having at least one comorbidity. The CG equation identified 21.6% of patients as having renal impairment compared with 17.6% with CKD-EPI[AS] and 9.3% with CKD-EPI[ASR]. There was a mortality rate of 9.1% across the study period. The highest mortality risk was seen in those with renal impairment as determined by the CKD-EPI[ASR] equation for both eGFR < 90 and eGFR < 60 with OR 2.97 (95%CI 1.86–4.76) and OR 10.6 (95%CI 3.15–18.04) respectively.

**Conclusion:**

In treatment naïve PWH in Zimbabwe, the CKD-EPI[ASR] equation identifies patients at highest risk of mortality when compared to the CKD-EPI[AS] and CG equations.

## Introduction

With increasing availability of antiretroviral therapy in Sub-Saharan Africa, the burden of disease in people living with HIV is shifting from infectious complications to non-infectious comorbidities. Chronic kidney disease (CKD) is of particular concern given its high prevalence and association with increased morbidity and mortality, even with early stages of CKD [1] Given the progressive nature of kidney disease, reducing its impact depends on its early diagnosis and treatment [2]. This is complicated, however, by a lack of consensus on how to appropriately monitor renal function in PWH from the Sub-Saharan region.

Gold standard measurements of GFR include inulin, iohexol and technetium labelled DTPA [2, 3]; however, assessments are expensive and cumbersome [4]. Consequently, a number of equations have been developed to estimate glomerular filtration rate using serum creatinine. Cockcroft-Gault (CG) was previously the most commonly used equation which estimated creatinine clearance (CrCl) [3]. The CG equation may be more accurate in estimating GFR in patients with malnutrition and low BMI [5, 6]. This may be of particular relevance to PWH who have been shown to have lower BMI compared to HIV negative patients [7].

The CG equation has been largely surpassed by the 2009 Chronic Kidney Disease Epidemiology Collaboration [Age, Sex, Race] (CKD-EPI[ASR]) which contains an adjustment for black race. The appropriateness of the race coefficient is being debated due to the potential risk of underdiagnosis of CKD in black patients [4]. This concern lead to the removal of the race coefficient from the equation and development of the 2021 CKD-EPI[Age and Sex] equation, which is now recommended for the calculation of eGFR in all American laboratories [8, 9]. Limited data from Sub-Saharan Africa also appear to support this change, with the removal of the race coefficient improving accuracy of estimates of GFR in the general population [10–12].

Studies validating eGFR equations in PWH in the Sub-Saharan African region are lacking, although estimates of renal impairment using these equations have been compared. In a large systematic review of PWH in Africa, CKD prevalence was found to be significantly lower at 7.0% using the CKD-EPI[ASR] equation compared to 13.7% when calculated by CG equation, with CKD defined as an eGFR < 60mL/min/1.73m^2^ [13]. Previous studies in PWH in Sub-Saharan Africa, including one in Zimbabwe, have shown a similar classification of severe renal impairment (eGFR < 30ml/min/1.73m^2^) when using the Modification of Diet in Renal Disease (MDRD) or CG Eq. [5]. However, they have shown the CG equation to identify a greater proportion of patients as having moderate renal impairment [5]. A comparison of eGFR equations in PWH in Ethiopia found a high correlation between equations, including CG and CKD-EPI with and without the race coefficient. However, when compared to 24 h urine creatinine excretion, CKD-EPI[AS] was found to have the highest accuracy [7].

Given the lack of validation studies of estimating equations in PWH from Sub-Saharan Africa, other factors need to be considered when selecting which to use. A logical consideration would be selecting that which identifies patients at highest risk of poor clinical outcomes. This may permit better targeting of limited healthcare resources. In this analysis we therefore compare CG, 2009 CKD-EPI[ASR] and 2009 CKD-EPI[AS] equations in a population of ART naïve PWH to assess which equation best predicts mortality.

## Methods

We conducted a retrospective cohort study of ART naïve patients commencing treatment for HIV at the Newlands Clinic, a charitable, not-for-profit, outpatient service focused on the management of PWH in Harare, Zimbabwe. This facility manages a high-burden of HIV and chronic kidney disease. Ethical approval was granted by the Medical Research Council of Zimbabwe (MRCZ/E/258). The study was conducted in accordance with the Declaration of Helsinki.

The study included all adult patients commencing ART at the clinic between January 2007 and September 2019. Patients were excluded from the study if no baseline creatinine measurement was available. Data were extracted from the clinic’s electronic medical records, including demographic information, hypertension and diabetes status, renal function indicators and BMI. BMI values were categorised into four groups, as defined by the World Health Organisation. Mortality events were either reported to the clinic by local hospitals or through notification by treatment buddies or next of kin. Proteinuria was measured using Combur10 Test UX (Roche, Switzerland) and read using a Urisys 1100 (Roche, Switzerland). Proteinuria was defined as ≥ 1 + on dipstick. Creatinine was measured by Reflotron Sprint (Roche, Switzerland) until 2015 and then by a COBAS Integra 400 Plus (Roche, Switzerland). Renal function was then calculated by the Cockcroft-Gault, CKD-EPI[ASR] and CKD-EPI[AS] equations for each patient. The CKD-EPI[AS] equation omitted the multiplier (eGFR x 1.159) for black race in the original CKD-EPI equation. Renal impairment was defined as an eGFR of less than 90ml/min/1.73m^2^, consistent with the Kidney Disease Improving Global Outcomes (KDIGO) classification of CKD, proteinuria was included as a separate variable in regression analysis.

Values are presented as mean (standard deviation) or median (interquartile range), as appropriate. Data were considered normal if both skewness and kurtosis values were between − 1 and 1. Categorical variables were compared by chi-square test. Comparisons between estimating equations were by the McNemar test, as the values generated by the equations were from the same cohort and thus related samples. Estimating equations were compared for patients by BMI group. Predictors of mortality were assessed by multivariable logistic regression of complete cases only, with the respective goodness-of-fits evaluated by their Akaike Information Criterion (AIC). To compare the magnitudes of the odds ratios (ORs) derived by the models, the differences in the natural logs of the ORs were used to calculate a z score that was then compared to a normal distribution to give the corresponding P value. Statistical analyses were preformed using SPSS v26 (IBM, USA).

## Results

There were 2991 patients included in the analysis. Patient characteristics are detailed in Table 1. The majority were female (62.1%), with a mean age of 38.9 years (SD 10.0). Approximately one fifth of patients had proteinuria at baseline (22.2%), but this persisted in less than half of affected patients after commencing ART (10.2%). Hypertension was common in the cohort (15.7%), whereas a history of diabetes was noted in only 2.4% of patients. Approximately one-in-eight patients (13.2%) had a BMI < 18.5 kg/m^2^. Including diabetes, hypertension, and baseline renal impairment, 26.1% of patients had at least one non-communicable disease at presentation.

With the CG equation, 21.6% of patients were classed as having baseline renal impairment (CrCl < 90 mL/min). This proportion fell to 17.6% with the CKD-EPI[AS] equation and to 9.3% with the CKD-EPI[ASR] equation (Table 2). Each of these estimates were significantly different from one another (P < 0.001). Severe baseline renal impairment (eGFR < 30ml/min) was rare, regardless of the equation used, with prevalence ranging from 0.6 to 1.2%.

**Fig. 1:**
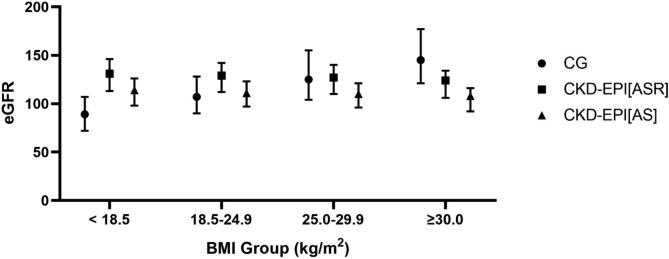
Comparison of the median eGFR between BMI groups by estimating equation. Error bars show interquartile range. Within each BMI group, the differences between estimating equation were statistically significant (p < 0.001 for all). BMI - body mass index, CG - Cockcroft-Gault, CKD-EPI - Chronic Kidney Disease Epidemiology Collaboration

Estimating equations are compared by BMI group in Fig. 1. The eGFR calculated by CG was closer to that estimated by the CKD-EPI[AS] equation in patients with a low or normal BMI. In overweight and obese patients, CG estimates were most comparable to the CKD-EPI[ASR] equation. The differences in eGFR as calculated by CG and its closest comparator are, expectedly, most pronounced at the extremes of BMI.

Over a median follow-up period of 4.6 years (interquartile range 2.7 to 6.9), there was a mortality rate of 9.1% and a further 12.1% had their care transferred to another clinic or were otherwise lost to follow-up. Potential predictors of mortality were assessed in complete cases by multivariable logistic regression, with a separate model for each estimating equation (Table 3). BMI data were unavailable for 1028 patients, of which 58 were also missing baseline proteinuria data, leaving 1963 complete cases for analysis. In each model, baseline renal impairment, proteinuria, diabetes, and BMI were significant predictors of mortality. There were no associations with sex or age and mortality risk. Across the models, the range in the ORs for proteinuria (2.47 to 2.69), diabetes (3.09 to 3.38) and BMI (0.92 to 0.93) were narrow, in contrast to baseline renal impairment.

A baseline eGFR < 90 mL/min/1.73m^2^ was associated with a nearly three-fold increased mortality risk when using the CKD-EPI[ASR] equation (OR 2.97, 95%CI 1.86 to 4.76, P < 0.001) and approximately doubled risk with the CKD-EPI[AR] equation (OR 2.14, SE 95%CI 1.42 to 3.23, P < 0.001). In contrast, a creatinine clearance calculated by CG of < 90 mL/min only increased mortality risk by 1.53-fold (95%CI 0.99 to 2.34, P = 0.049). Comparing AIC values, CKD-EPI[ASR] had the best fit overall and was significantly preferred to both the CG (P < 0.001) and CKD-EPI[AR] (P = 0.049) equations. CKD-EPI[AS] was also preferred to CG (P = 0.016).

Using an eGFR of < 60 to define baseline renal impairment resulted in an increased risk of mortality for all estimating equations(P < 0.001 for all): CG (OR 3.57, 95%CI 1.53 to 5.61), CDK-EPI[ASR] (OR 10.6, 95%CI 3.15 to 18.04), and CKD-EPI[AS] (OR 5.63, 95%CI 2.24 to 8.96). The AIC for the models were 839.1, 815.2 and 827.5, respectively. Again, CKD-EPI[ASR] was significantly preferred to CG (P < 0.001) and CKD-EPI[AS] equations (P = 0.002) and CKD-EPI[AS] was preferred to CG (P = 0.003).

**Table 1:** Patient characteristics by survival status (N = 2991)

Variable	All Patients (%)	Surviving (%) [N = 2179]			Deceased (%) [N = 272]	P-value*
Sex						< 0.001
Female	1857 (62.1)	1728 (79.3)			129 (47.4)	
Male	1134 (37.9)	991 (20.7)			143 (52.6)	
Age group (N, %)						< 0.001
18–29 years	583 (19.5)	544 (25.0)			39 (14.3)	
30–39 years	1047 (35.0)	976 (44.8)			71 (26.1)	
40–49 years	899 (30.1)	812 (37.3)			87 (32.0)	
50–59`years	321 (10.7)	274 (12.6)			47 (17.3)	
≥ 60 years	141 (4.7)	113 (5.2)			28 (10.3	
BMI Category [N = 1963]						< 0.001
< 18.5	260 (13.2)	226 (12.5)			34 (23.0)	
18.5–24.9	1039 (52.9)	956 (52.7)			83 (56.1)	
25.0- 29.9	393 (20.0)	378 (20.8)			15 (9.1)	
≥ 30	271 (13.8)	255 (14.0)			16 (10.8)	
Baseline Proteinuria [N = 2942]	653 (22.2%)	502 (23.0)			151 (55.5)	< 0.001
Persistent proteinuria	300 (10.2%)	267 (12.3)			33 (12.1)	0.226
Hypertension	469 (15.7%)	432 (19.8)			37 (13.6)	0.124
Diabetes	71 (2.4%)	60 (2.8)			11 (4.0)	0.058

BMI - body mass index (kg/m^2^); *comparison between surviving and deceased patients by Chi-squared test.

**Table 2:** Distribution of baseline renal function by estimating equation

eGFR	CG	CKD-EPI[ASR]	CKD-EPI[AS]
**≥ 90**	2345 (81.4%)	2714 (90.7%)	2466 (82.4%)
**60–89**	503 (16.8%)	207 (6.9%)	416 (13.9%)
**45–59**	75 (2.5%)	29 (0.8%)	57 (1.9%)
**30–44**	31 (1.0%)	21 (0.7%)	24 (0.8%)
**15–29**	27(0.9%)	13 (0.4%)	20 (0.7%)
**< 15**	10 (0.3%)	7 (0.2%)	8 (0.3%)

CG - Cockcroft-Gault, CKD-EPI - Chronic Kidney Disease Epidemiology Collaboration, eGFR - estimated glomerular filtration rate (mL/min for CG and mL/min/1.73m^2^ for CKD-EPI).

**Table 3:** Predictors of mortality in multivariable logistic regression by eGFR estimating equation

	Variable	Odds Ratio	95%CI	P-value	AIC
**CG**	**Male**	0.97	0.66 to 1.44	0.882	852.4
	**Age**	1.01	0.99 to 1.03	0.229	
	**eGFR < 90**	1.53	0.99 to 2.34	0.049	
	**Proteinuria**	2.69	1.85 to 3.91	0.000	
	**Diabetes**	3.26	1.37 to 7.71	0.008	
	**BMI**	0.93	0.90 to 0.97	0.002	
**CKD-EPI[ASR]**	**Male**	0.98	0.66 to 1.45	0.912	838.1
	**Age**	1.01	0.99 to 1.03	0.510	
	**eGFR < 90**	2.97	1.86 to 4.76	0.000	
	**Proteinuria**	2.47	1.66 to 3.64	0.000	
	**Diabetes**	3.09	1.28 to 7.48	0.012	
	**BMI**	0.92	0.89 to 0.96	0.000	
**CKD-EPI [AS]**	**Male**	0.95	0.64 to 1.41	0.806	844.1
	**Age**	1.01	0.99 to 1.03	0.381	
	**eGFR < 90**	2.14	1.42 to 3.23	0.000	
	**Proteinuria**	2.59	1.78 to 3.75	0.000	
	**Diabetes**	3.38	1.43 to 8.02	0.006	
	**BMI**	0.92	0.88 to 0.95	0.000	

BMI - body mass index (kg/m^2^), CG - Cockcroft-Gault, CKD-EPI – Chronic Kidney Disease Epidemiology Collaboration, eGFR - estimated glomerular filtration rate (mL/min for CG and mL/min/1.73m^2^ for CKD-EPI)

## Discussion

In a large cohort of PWH from Zimbabwe, the prevalence of renal impairment (eGFR or creatinine clearance < 90) ranged from 9.7 to 18.6%, using the CKD-EPI[ASR] and CG equations respectively. Individuals with renal impairment defined by the CKD-EPI[ASR] equation had a three-fold higher mortality risk than those without renal impairment. In contrast, renal impairment defined by the CG equation was only associated with a 1.5-fold increased mortality risk.

The prevalence of renal impairment (eGFR < 90) in Zimbabwe has been reported to range between 3.1 and 30.2% dependent on the population studied and use of proteinuria or serum creatinine to identify renal impairment [5, 14]. The prevalence in our cohort was higher than that reported by Stohr et al. in 2008 who reported a rate of 3.1–7.4% using CG and MDRD formulae to estimate GFR in patients from a similar population in Hanare, Zimbabwe [5]. The different prevalence of renal impairment may be explained by cohort differences, however, an increasing rate of non-communicable diseases including diabetes and hypertension in PWH may contribute to increasing development of renal impairment [14]. Patient characteristics were similar to those reported in recent Zimbabwean studies of PWH, with comparable rates of diabetes and hypertension, suggesting generalisability of our outcomes [14–16].

Omission of the race coefficient from the CKD-EPI[ASR] equation approximately doubled prevalence of renal impairment (eGFR < 90 mL/min/1.73m^2^) within our cohort at 9.3 vs. 17.6%. This result is similar to that reported in a retrospective analysis of Black American patient cohort data, which found that using the 2021 CKD-EPI[AS] equation increased the prevalence of patients with eGFR < 60mL/min/1.73m^2^ from 11 to 15% [9]. In our study, the increased identification of renal impairment using the 2009 CKD-EPI[AS] equation was primarily in patients with mild renal impairment (eGFR 60–90 mL/min/1.73m^2^). These findings are in keeping with previous criticism of the CKD-EPI[ASR] equation and its reduced identification of early CKD in Black patients [8].

Defining renal impairment as an eGFR < 60, as compared to < 90, approximately doubled the associated mortality risk. This trend has been similarly demonstrated in cohorts of PWH from Ghana and Zambia [6, 17]. Comparing the two CKD-EPI equations, the difference in mortality prediction is primarily driven by the eGFR equation, with similar odds ratio for mortality from other predictive factors including diabetes, proteinuria and BMI in each model. These factors have all been shown to be associated with increased mortality in PWH [6, 18, 19]. In all multivariable models the greatest predictors for mortality apart from eGFR were proteinuria and diabetes with OR ranging between 2.5 and 3.4, similar to previous analysis [20, 21].

With changes in body composition at the extremes of BMI, estimation of GFR is more challenging. When compared to gold standard, CG and CKD-EPI have similar accuracy at low BMI while CKD-EPI is more accurate at high BMI [22]. We have found a similar result with comparable estimates of GFR using CG and CKD-EPI equations in patients with low and normal BMI. However, CG estimated significantly higher GFR in patients with a high BMI, this is likely an overestimate as the CG equation includes a weight variable which does not discern between adipose tissue and creatinine producing muscle [5]. As expected in our predominantly Black African population, the removal of the race coefficient resulted in lower estimates of GFR in all BMI groups.

In multivariable analysis, identification of renal impairment using the CKD-EPI[ASR] equation was associated with the highest mortality rate. The higher OR for mortality using CKD-EPI[ASR] may be explained by a lower number of patients classified as mild renal impairment, in whom few deaths occurred. Interestingly, the difference in mortality persists when using an eGFR of < 60ml/min/1.73m^2^ to define renal impairment. A similar result of mortality assessment with the CKD-EPI[ASR] equation was found be Gutiérrez et al. in a large American retrospective cohort study involving 62,011 patients with 33% of patients identifying as Black. In this study the CKD-EPI[ASR] and 2021 CKD-EPI[AS] equations showed similar hazard ratios (HR) for all-cause and cardiovascular mortality, though CKD-EPI[ASR] predicted higher HR for both outcomes [9].

The main limitation of our study was the use of single measures of creatinine to define baseline renal impairment, despite serum creatinine being known to fluctuate [2]. In a resource-limited settings, repeated measures may not be feasible. That we have found a robust relationship between renal impairment and mortality using single measures alone, suggests this method of assessment is still useful in identifying patients at greater mortality risk. A second limitation is that this study only included patients from a single, urban centre and therefore may not be as generalisable to the wider PWH population. The short duration of follow-up and large percentage of patients lost to follow up are further limitations, however, this patient cohort is at high risk of premature mortality with a rate of almost 10% across the study period. A large proportion of patients did not have BMI data and were excluded from regression models, this was largely due to height not being recorded which likely occurred at random and is therefore unlikely to impart significant bias in the analysis though does reduce the power and precision of the estimates. Finally, the inclusion of HIV viral load, CD4 counts and response to HIV therapy would be important to include in future studies to assess their contribution to mortality.

## Conclusion

ART naïve patients with baseline renal impairment, as defined by the CKD-EPI[ASR] equation, had a significantly higher risk of mortality than patients with renal impairment defined be either the CKD-EPI[AS] or CG equations. While validation studies are pending, this capacity to identify patients at highest clinical risk may make CKD-EPI[ASR] the superior equation for evaluating renal function in resource-limited settings.

## Data Availability

All data generated or analysed during this study are included in this published article.

## List of Abbreviations

BMI: Body Mass Index
CG: Cockcroft-Gault (CG)
CKD: Chronic Kidney Disease
CrCl: Creatinine Clearance
CKD-EPI[ASR]: Chronic Kidney Disease Epidemiology Collaboration Equation
CKD-EPI[AS]: CKD-EPI equation with the race coefficient removed
eGFR: Estimated Glomerular Filtration Rate
PWH: People living with HIV
